# Single-Ascending-Dose Pharmacokinetic Study of Tribendimidine in Opisthorchis viverrini-Infected Patients

**DOI:** 10.1128/AAC.00992-16

**Published:** 2016-09-23

**Authors:** Urs Duthaler, Somphou Sayasone, Fiona Vanobbergen, Melissa A. Penny, Peter Odermatt, Jörg Huwyler, Jennifer Keiser

**Affiliations:** a Swiss Tropical and Public Health Institute, Basel, Switzerland; b University of Basel, Basel, Switzerland; c National Institute of Public Health, Ministry of Health, Vientiane, Lao People's Democratic Republic; d Department of Pharmaceutical Sciences, Division of Pharmaceutical Technology, University of Basel, Basel, Switzerland

## Abstract

Praziquantel is the only drug available for the treatment of Opisthorchis viverrini infections. Tribendimidine has emerged as a potential treatment alternative; however, its pharmacokinetic (PK) properties have not been sufficiently studied to date. Via two phase IIa dose-finding studies, 68 O. viverrini patients were treated with 25- to 600-mg doses of tribendimidine using 50- and 200-mg tablet formulations. Plasma, blood, and dried blood spots (DBS) were sampled at selected time points. The two main metabolites of tribendimidine, active deacetylated amidantel (dADT) and acetylated dADT (adADT), were analyzed in plasma, blood, and DBS. PK parameters were estimated by noncompartmental analysis. An acceptable agreement among plasma and DBS concentrations was observed, with a mean bias of ≤10%, and 60% dADT and 74% adADT concentrations being within ±20% margins. We found that 200-mg tribendimidine tablets possess immediate floating characteristics, which led to variable time to maximal concentration of drug (*T*_max_) values (2 to 24 h) between individuals. Dose proportionality was observed for dADT from 25 to 200 mg using 50-mg tablets, but at higher dosages (200 to 600 mg), saturation occurred. The median ratio of the area under the plasma concentration-time curve from 0 to 24 h (AUC_0–24_) of dADT to the AUC_0–__24_ of adADT ranged from 0.8 to 26.4, suggesting substantial differences in acetylation rates. Cure rates ranged from 11% (25-mg dose) to 100% (400-mg dose). Cured patients showed significantly higher dADT maximal serum concentrations (*C*_max_) and AUC_0–24_ values than uncured patients. Tribendimidine is a promising drug for the treatment of opisthorchiasis. However, the tablet formulation should be optimized to achieve consistent absorption among patients. Further studies are warranted to assess the large differences between individuals in the rate of metabolic turnover of dADT to adADT. (This study has been registered with the ISRCTN Registry under no. ISRCTN96948551.)

## INTRODUCTION

Approximately 45 million people are infected with the opisthorchiid flukes Clonorchis sinensis, Opisthorchis felineus, and O. viverrini. O. viverrini predominantly occurs in the Lower Mekong Basin in north and northeast Thailand, Lao People's Democratic Republic, Cambodia, and southern Vietnam, where it represents a significant health problem (1–3). Infection with O. viverrini can lead to hepatobiliary disease, including hepatomegaly, cholangitis, cholecystitis, fibrosis of the periportal system, and gallstones. However, the most serious complication is its involvement in the development of human cholangiocarcinoma (CCA), which has a poor prognosis (4).

A vaccine is presently not available to prevent opisthorchiid infections, and praziquantel is currently the only recommended drug for the treatment of infections with O. viverrini. The drug is widely used in preventive chemotherapy programs aiming to reduce disease-associated morbidity. Although praziquantel is an efficacious drug, adverse events are common (5–8). Furthermore, development of drug resistance is a risk if only a single drug is used for the treatment and control of this important liver fluke infection.

Tribendimidine is a broad-spectrum anthelmintic agent developed and approved in China for the treatment of soil-transmitted helminth infections (9), and it also has excellent activity against the liver flukes C. sinensis and O. viverrini, as demonstrated in two exploratory randomized trials (8, 10). We therefore recently started a clinical development program on tribendimidine for the treatment of O. viverrini infections consisting of a dose-finding phase IIa trial and a IIb trial in Lao People's Democratic Republic. Pharmacokinetic (PK) studies are an essential part of drug discovery and development. PK parameters can be influenced by disease, and hence, data from healthy volunteers are not always predictive of the patient population. For example, PK parameters of praziquantel were shown to be considerably different in the moderately advanced stage of opisthorchiasis compared to those during asymptomatic infections, likely due to an impairment of the metabolism (11). To date, PK studies with tribendimidine have been conducted only in healthy volunteers (12). It was shown that after drug dissolution, tribendimidine decomposes immediately into two deacetylated amidantel (dADT) moieties and the terephthalaldehyde linker. Tribendimidine itself cannot be detected in the circulatory system. dADT reaches maximal plasma concentrations at ∼4 h postdosing and has a half-life of about 4 h. dADT is partially acetylated to acetylated dADT (adADT), most likely by arylamine *N*-acetyltransferases (NAT-1 and NAT-2), and about one-third of dADT is excreted unchanged through the urine (12).

To facilitate PK studies in O. viverrini-infected patients, we recently developed a liquid chromatography-tandem mass spectrometry (LC-MS/MS) method for the determination of dADT and adADT levels in plasma, blood, and dried blood spots (DBS) (13). The DBS technique is a practical microsampling tool to overcome the difficulties of PK sample collection, storage, and shipment in remote settings where worm infections commonly occur (14). Thereby, venous puncture is replaced by less invasive pricking of the finger pulp. Subsequently, capillary blood is dropped onto filter paper, dried, and stored at ambient temperature. Uniform diffusion of the blood on the filter paper allows quantitation based on the area of the blood spots (15).

The present study aimed to identify PK profiles of ascending doses of tribendimidine in plasma, blood, and DBS in patients infected with O. viverrini. Drug dissolution experiments were included to obtain a better understanding of the PK properties of tribendimidine. Moreover, drug exposure and treatment outcome were compared to shed first light into the PK/pharmacodynamic (PD) relationship of tribendimidine against opisthorchiasis.

## MATERIALS AND METHODS

### Study design and ethical considerations.

This PK study in O. viverrini-infected patients was embedded in two randomized, single-blind, phase IIa, dose-escalation trials (16), which were conducted from September 2012 to December 2013. In the first trial (study 1), O. viverrini-infected participants received 200 mg, 400 mg, or 600 mg using 200-mg tribendimidine tablets. In the second trial (study 2), patients were treated with 25 mg (50-mg tablet cut in half), 50 mg, 100 mg, and 200 mg (four 50-mg tablets) using 50-mg tablets (16).

Ethical approval for the trials was received from the National Ethic Committee for Health Research, Ministry of Health (MoH), Lao People's Democratic Republic (reference no. 009/NECHR); the ethics committee of Northern and Central Switzerland, Switzerland (EKNZ) (reference no. 375/11); and the Ethic Committee of the Liverpool School of Tropical Medicine, Liverpool, United Kingdom (reference no. 12.02RS). Permission was also obtained from the Ministry of Health, the Provincial Health Office, and the District Health Office in Lao People's Democratic Republic. All patients gave written, informed consent prior to undergoing any study-related procedures. The study has been registered with the ISRCTN Registry (no. ISRCTN96948551). All participants diagnosed with a helminth infection at the end of the study were offered anthelminthic treatment (praziquantel and albendazole) according to national guidelines.

### Patients, treatment, and study procedures.

O. viverrini-infected adult patients, diagnosed during baseline screening for the phase IIa trials (16), were invited to participate in the PK study and admitted to Champasack Provincial Hospital, Pakse, Lao People's Democratic Republic, for 24 h. Medical history was assessed with a standardized questionnaire and a full clinical examination, including measurement of weight, height, and blood pressure. Female participants underwent pregnancy testing using a urine sample. Eligible patients were those who did not suffer from major systemic or chronic illnesses and psychiatric and neurological disorders and who were not pregnant.

Stool samples were taken prior to treatment and 21 to 23 days later (16) to estimate egg burden before and after treatment in order to quantify the PD effect of tribendimidine against O. viverrini. At each of these time points, two stool samples were collected on different days within a maximum of 3 days. Two slides were prepared from each stool sample and analyzed by using the Kato-Katz technique (17). Cure rates were calculated as the percentage of participants who became egg negative after treatment. The infection intensity (number of eggs per gram [epg] of feces) was assessed by adding the individual egg counts from quadruplicate Kato-Katz thick smears and multiplying this value by 6. Arithmetic mean egg counts were calculated for each group before and after treatment. Egg reduction rates (ERRs) were calculated by using the following formula: ERR = [1 − (arithmetic mean at follow-up/arithmetic mean at baseline)] × 100.

Fifty-milligram and 200-mg tribendimidine tablets with enteric coating were purchased from Shandong Xinhua Pharmaceutical Co., Ltd. (Zibo, People's Republic of China). Drugs were administered in the presence of an investigator after providing a standardized food item to the participant.

Approximately 5 ml blood was withdrawn by an intravenous catheter (during PK sampling) to determine the renal and hepatic functions of the participants pretreatment (azotemia [milligrams per deciliter], creatinine [milligrams per deciliter], aspartate aminotransferase [ASAT]-glutamate oxaloacetate transaminase [GOT] [units per liter], and alanine aminotransferase [ALAT]-GOT [units per liter] levels). Creatinine clearance was estimated based on serum creatinine levels by using the Chronic Kidney Disease Epidemiology Collaboration equation (18).

Venous blood sampling was performed at ∼0, 1, 2, 3, 4, 4.5, 5, 6, 8, 10, and 24 h posttreatment on the basis of previously reported tribendimidine PK data (12). DBS were collected for half of the participants at ∼0, 1, 3, 4.5, 6, and 10 h, while the other half were sampled at ∼0, 2, 4, 5, 8, and 24 h. Venous blood (4 ml) was collected in EDTA-covered Vacutainer tubes (BD, Switzerland) through an intravenous catheter placed into an antecubital arm vein. For the purpose of keeping the catheter patent, 0.9% sodium chloride (NaCl) was used for rinsing. Dilution artifacts were avoided by discarding the first 0.5 ml of blood. Immediately after the collection of the required blood volume, the Vacutainer tubes were slowly tilted backwards and forwards to dissolve the anticoagulant. One milliliter of blood was transferred into a cryotube within 15 min postsampling. The remaining blood in the Vacutainer was centrifuged at about 3,000 rpm for 10 min to obtain plasma, which was transferred into a second cryotube. Plasma and blood tubes were kept in an upright position at −20°C until analysis. Capillary blood (0.1 ml) was collected by middle or ring finger tip puncture using a finger pricker (Accu-chek Safe-T-Pro Plus; Roche Diagnostics, Rotkreuz, Switzerland). A few drops of blood were transferred onto filter paper (Whatman DMPK-C cards; GE Healthcare, Buckinghamshire, United Kingdom) by using capillaries and dried overnight. The DBS cards were stored at room temperature until analysis. Plasma and DBS measurements were performed in both studies. Blood measurements were performed in only the first study using 200-mg tablets since blood values were shown to agree with plasma values (data from this study) (see Results).

### LC-MS/MS analysis.

dADT and adADT concentrations were analyzed in plasma, blood, and DBS samples by using a recently developed and validated LC-MS/MS method (13). In brief, analysis was performed on a quaternary high-performance liquid chromatography (HPLC) system (Shimadzu, Kyoto, Japan) coupled to an API 3000 tandem mass spectrometer (AB Sciex, MA, USA). A core shell pentafluorophenyl column (Kinetex PFP column [2.6-μm particle size, 100 Å, and 50 by 4.6 mm]; Phenomenex, Torrance, CA, USA) was used for chromatography of the analytes. Separation was achieved within 2.5 min by using an isocratic elution of 10% mobile phase A (0.15% formic acid, 10 mM ammonium acetate in water)–90% mobile phase B (0.15% formic acid plus acetonitrile and 100 mM aqueous ammonium acetate [90:10, vol/vol]). dADT (*m/z* 178.3 → 133.1) and adADT (*m/z* 220.4 → 175.1) and its deuterated internal standards dADT-d6 (*m/z* 184.1 → 133.1 *m/z*) and adADT-d6 (*m/z* 226.1 → 175.1) were detected by multiple-reaction monitoring in the positive ionization mode. A lower limit of quantification (LLOQ) of 1 ng/ml dADT and adADT was realized in plasma and blood. A sensitivity of 10 ng/ml in DBS in study 1 was improved (1 ng/ml) by punching out two 3-mm-diameter discs per blood spot in study 2. Extraction was carried out with acetonitrile (plasma) or a mixture of acetonitrile and mobile phase A (blood, 70%–30% [vol/vol]; DBS, 50%–50% [vol/vol]). At least eight calibrators (plasma and blood, 1 to 1,000 ng/ml; DBS, 10 to 2,000 ng/ml), a double-blank sample (matrix processed without an internal standard), and a blank sample (matrix processed with the internal standard) were prepared on each day of analysis. To monitor the quality of the analyses, six replicates of quality control (QC) samples at the LLOQ and low, medium, and high concentrations were included in each analytical run. In the case of DBS QC samples, different hematocrits were prepared based on the magnitude observed in the study population (study 1, 20 to 45%; study 2, 25 to 50%). A hematocrit of 35% was chosen for calibrators, which is approximately the median hematocrit in both study populations, to diminish biases caused by variable hematocrits. A precision of 15% (LLOQ, 20%) and a mean accuracy of 85 to 115% (LLOQ, 80 to 120%) were accepted in this study. Interassay accuracy and precision were calculated over all analytical runs.

### Drug dissolution experiments.

Drug dissolution of 200 mg and 50 mg tribendimidine tablets with enteric coating was studied with a USP-2 (U.S. Pharmacopeia) dissolution apparatus (Sotax AT7; Sotax, MA, USA). A continuous circulatory flow between the six vessels of the USP-2 apparatus and the UV detector (Amersham Ultrospec 3100; GE Healthcare) was generated by using a peristaltic pump (Sotax CY7; Sotax, MA, USA). The pump was set at 50 rpm, and every fifth minute, the UV absorption was measured at 260 nm. The stirrer within the vessels was set at 100 rpm.

During the first 2 h of each experiment, the tablets (*n* = 3 per experiment) were kept at pH 1.2 (1 liter HCl, 0.1 mol/liter per vessel according to Pharmacopeia Europaea II). Tablets were considered to be enterally stable if <5% of drug was released within this period. Subsequently, tablets were incubated in phosphate buffer solution adjusted to pH 6.8 (1 liter per vessel according to Pharmacopeia Europaea 7.7) until drug dissolution was completed. Calibration lines (12.5 to 200 mg/liter tribendimidine) were recorded at 260 nm in 0.1 M HCl and phosphate buffer to determine the quantity of drug release. Total tribendimidine release was quantified by measuring the absorption of tribendimidine and its degradation products.

### Pharmacokinetic and statistical analyses.

Secondary PK parameters of dADT and adADT were determined by noncompartmental analysis using WinNonlin (version 5.2; Certara, Princeton, NJ, USA). Maximal concentration of drug (*C*_max_) (nanograms per milliliter) and time to maximal concentration of drug (*T*_max_) (hours) were observed values. When three or more observations in the elimination phase were available, the elimination half-life (*t*_1/2_) was calculated as *t*_1/2_ = ln(2)/λ, where the elimination rate constant of the analyte (λ) was determined by linear regression of the natural logarithm of the concentration values. The area under the plasma concentration-time curve (AUC) (nanograms times hour per milliliter) and the area under the first-moment curve (AUMC) (nanograms times hour squared per milliliter) were estimated from time point zero to the time point of the last quantifiable concentration (AUC_0–24_ and AUMC_0–24_) or from time point zero extrapolated to infinity (AUC_∞_ and AUMC_∞_) by using the linear trapezoidal rule. The mean residence time (MRT) was calculated as the AUMC_∞_/AUC_∞_ ratio. Drug clearance (CL/*F*) was calculated for dADT as the dose divided by the AUC_∞_, assuming that tribendimidine is completely converted into two dADT molecules. The dose was calculated based on the molecular weight of dADT. Samples below the LLOQ were estimated as LLOQ/2 (0.5 ng/ml) (19). PK parameters were estimated for each study participant, and additionally, the median and the interquartile range (IQR) were calculated for each dosing cohort. For DBS data, only AUC_0–24_ was included due to the more limited sampling schedule.

Statistical analyses were conducted with GraphPad Prism 6.01 (GraphPad, CA, USA) and Stata version 12 (StataCorp, College Station, TX). Mann-Whitney statistical tests were used to compare PK parameters by dose. A *P* value of <0.05 was considered to be statistically significant.

Dose proportionality was assessed by using a power model and a criterion based on comparing the 90% confidence interval (CI) for the ratio of dose-normalized means to the prespecified limits of 0.5 to 2, as proposed previously by Hummel et al. (20). In this study, this equated to performing a regression of log AUC_∞_ against log dose and concluding equivalence if the 90% CI for the slope lay within 0.78 to 1.22. We further considered adjusting for weight and tablet formulation (50 mg and 200 mg) and an interaction between log dose and tablet.

Data obtained from plasma, blood, and DBS measurements were compared by using Bland-Altman analysis to assess the level of agreement between biofluids (21, 22). The measured concentration and estimated AUC_0–24_ data for the different fluids were compared pairwise by plotting the percent differences {% difference = [(concentration in fluid 1 − concentration in fluid 2)/mean concentration] × 100} against their mean values. Only concentration data that were available for both biofluids were included. The mean deviation and the 95% limits of agreement (±2 times the standard deviation) were assessed. Moreover, concentrations measured in the different study sample matrices were cross-validated according to bioanalytical method validation guidelines, whereby the percent difference of the two values obtained should be within 20% for at least 67% of the repeats (23, 24).

AUC_0–24_, AUC_∞_, and *C*_max_ ratios of dADT to adADT were determined for each patient in order to investigate the intersubject variations in the acetylation rate catalyzed by NAT-1/2. The associations between the AUC_0–24_, AUC_∞_, and *C*_max_ ratios of dADT to adADT and cure were assessed by using univariable logistic regression. Only tribendimidine doses (100 to 600 mg) that were demonstrated to be efficacious against O. viverrini were included in this analysis (16).

## RESULTS

### Participant characteristics and treatment efficacy.

A total of 68 individuals were enrolled in the PK study (study 1, 200 to 600 mg using 200-mg tablets; study 2, 25 to 200 mg using 50-mg tablets). Treatment groups consisted of 9 to 13 patients each (Table 1). Overall, 51% of the participants were female, the age ranged between 15 and 65 years (median, 42 years), and the median body weight was 52 kg. Sex, age, and body weight were broadly comparable across treatment groups (Table 1). Azotemia levels and creatinine clearance indicate that preexisting mild kidney dysfunction was present in several participants. In particular, treatment groups receiving 50, 100, and 200 mg (study 2) displayed low creatinine clearance values. Elevated liver enzyme levels were observed in 47% of the participants.

**TABLE 1:** Participant characteristics

Characteristic	Value for group							
	2nd study (50-mg tablets) (*n* = 37)				1st study (200-mg tablets) (*n* = 31)			Total
	25 mg	50 mg	100 mg	200 mg	200 mg	400 mg	600 mg	
Demographics								
No. of participants	9	9	9	10	13	9	9	68
No. (%) of female patients	3 (33)	6 (67)	5 (56)	5 (50)	6 (46)	5 (56)	5 (56)	35 (51)
Median age (yr) (IQR)	45 (39–56)	42 (26–44)	44 (34–51)	39 (26–40)	44 (41–56)	37 (29–42)	43 (34–45)	42 (32–47)
Median wt (kg) (IQR)	51 (49–60)	48 (42–54)	47 (44–54)	55 (49–58)	54 (49–59)	49 (48–51)	53 (46–58)	52 (47–57)
Baseline liver and kidney function								
Median creatinine clearance (ml/min per 1.73 m^2^) (IQR)	94 (77–112)	49 (43–56)	53 (46–56)	48 (47–63)	108 (75–135)	108 (78–116)	113 (52–131)	66 (50–112)
Median azotemia level (mg/dl) (IQR)	29 (20–32)	27 (26–29)	29 (28–31)	30 (29–30)	24 (20–30)	21 (16–25)	20 (18–20)	27 (20–30)
Median ASAT-GOT level (U/liter) (IQR)	36 (29–39)	37 (34–54)	40 (32–40)	38 (29–39)	35 (27–39)	34 (30–40)	42 (34–44)	37 (29–41)
Median ALAT-GOT level (U/liter) (IQR)	37 (29–38)	39 (30–51)	27 (26–34)	34 (29–40)	24 (18–29)	23 (19–30)	28 (21–43)	29 (23–39)
Infection intensity and treatment activity								
Median egg burden at day 0 (epg) (IQR)	414 (384–432)	2,214 (1,224–3,024)	612 (504–774)	588 (461–777)	1,410 (648–1,914)	978 (204–1,476)	3,294 (1,848–5,256)	897 (437–1,817)
Median egg burden at day 21 (epg) (IQR)	54 (24–216)	78 (6–192)	0 (0–12)	0 (0–6)	3 (0–29)	0 (0–0)	0 (0–6)	0 (0–45)
Egg reduction rate (%)^*a*^	73.7	93.6	96.7	98.0	98.6	100	99.1	100 (93–100)
No. (%) of cured individuals	1 (11)	1 (11)	6 (67)	6 (60)	7 (54)	9 (100)	6 (66)	36 (53)

a Based on arithmetic means.

O. viverrini infection intensities were greater in the first study population than in the second study population. Overall, high egg reduction rates were observed 21 to 23 days after treatment, in particular at dosages of 100 mg and above. Cure rates with 25-mg to 600-mg doses ranged from 11 to 100%, with cure being observed in all patients treated with 400 mg.

### LC-MS/MS method.

The calibration lines measured in the three different matrices exhibited a correlation coefficient (*r*^2^) of >0.99. Any blank sample of the study participants showed matrix interferences. The interassay precisions of dADT (range, 2.9 to 10.4%) and adADT (range, 2.4 to 15.7%) were lower than the prespecified limit of 15% or 20% at the LLOQ (see Table S1 in the supplemental material). The interassay accuracy level ranged between 94.6 and 108.1%, taking all QC levels and matrices into account, and therefore was within acceptable bounds.

### Agreement between plasma, blood, and DBS results.

Overall, 285 dADT and 260 adADT concentrations were analyzed in both whole blood and plasma (study 1), and 287 dADT and 251 adADT concentrations were analyzed in both plasma and DBS (studies 1 and 2, with DBS being obtained at limited time points). Fewer adADT than dADT samples could be quantified because adADT reached lower systemic levels, and the method was equally sensitive for both analytes.

The percent differences in dADT concentrations in blood and plasma were consistent visually with no trend across individual concentrations or AUC_0–24_ (see Fig. S1 in the supplemental material). For adADT, the percent differences in individual concentrations and AUC_0–24_ tended to decrease slightly at higher values. Blood versus plasma concentrations indicated only a small bias (−12% for dADT and +6% for adADT) (see Fig. S1 in the supplemental material). Comparable 95% limits-of-agreement ranges were obtained for concentration ratios for both analytes (dADT, −33% to +9%; adADT, −10% to +22%). Overall, 78% of the dADT and 95% of the adADT concentrations exhibited a percent difference of ≤20%. For AUC_0–24_ values, the blood-versus-plasma bias was consistent with the concentration data, whereas 95% limits of agreement were narrower than those obtained for concentration data.

**FIG 1:**
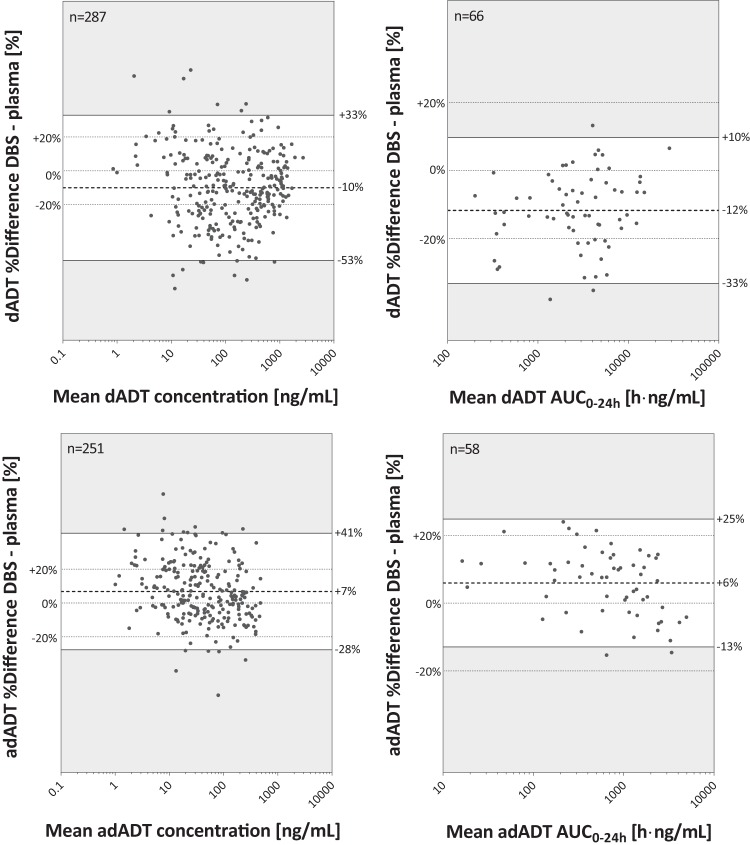
Bland-Altman plots of concentrations and AUC_0–24_ values measured in DBS and plasma samples (percent difference versus the mean). The AUC was calculated from time zero to the time point of the last quantifiable concentration. Only concentration data that were available for both biofluids were included. The dashed line in dark gray illustrates the mean percent difference of DBS compared to plasma. The white area defines the 95% limits of agreement. The ±20% biases from zero are marked in the plots, as they refer to limits applied for cross-validation of two bioanalytical methods. Gray dots correspond to observed values.

The percent difference in dADT concentrations of DBS and plasma did not show a trend across individual concentrations or AUC_0–24_ values (Fig. 1). However, for adADT, the percent difference tended to decrease at higher values, comparable to those for blood and plasma comparisons. The mean bias of dADT and adADT concentrations for DBS versus plasma was similar to those for comparisons of blood versus plasma (dADT, −10%; adADT, +7%). However, the 95% limits of agreement were wider (dADT, −53% to +33%; adADT, −28% to +41%). The percentages of dADT and adADT samples with a ≤20% difference were 60% and 74% for dADT and adADT, respectively; hence, adADT passed the criterion of 67% for cross-validation, while dADT did not. Agreement plots using AUC_0–24_ data showed biases similar to those in the case of plots using concentrations but with narrower 95% limits of agreement. Consequently, 79% of the dADT and 93% of the adADT AUC_0–24_ DBS-to-plasma ratios exhibited a percent difference of ≤20%.

### Pharmacokinetics of plasma and blood measurements.

**FIG 2:**
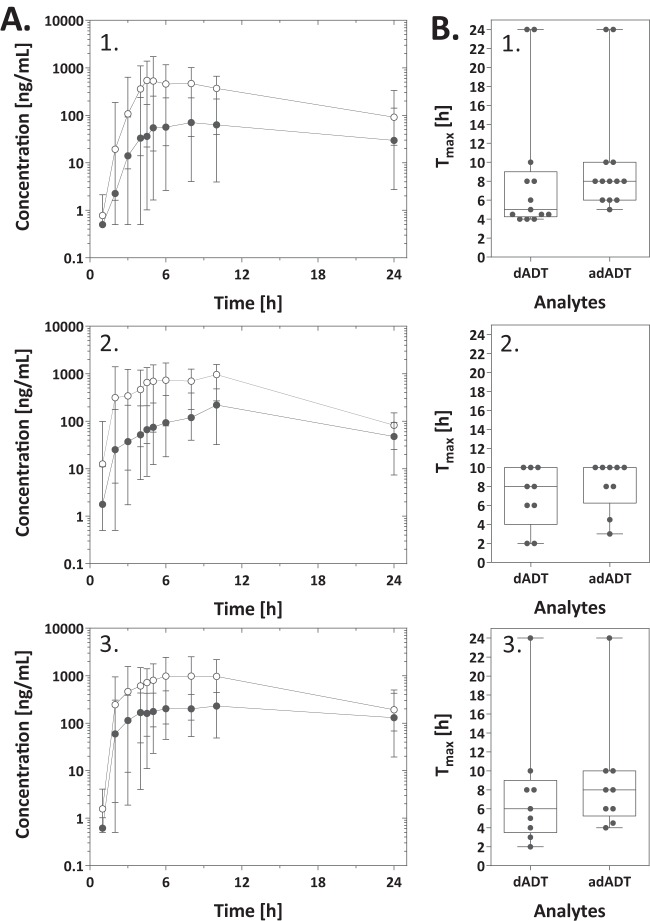
(A) Mean plasma concentration-time profiles for dADT (○) and adADT (●) following oral doses of one 200-mg tablet (*n* = 13) (A1), two 200-mg tablets (*n* = 9) (A2), and three 200-mg tablets (*n* = 9) (A3) of tribendimidine. The mean concentrations and ranges are illustrated. (B) Box plots of the *T*_max_ values for dADT and adADT (one 200-mg [B1], two 200-mg [B2], and three 200-mg [B3] tablets of tribendimidine). The upper and lower limits of the boxes correspond to the interquartile range, the value in the middle corresponds to the median, and the limits of the whiskers correspond to the range. Gray dots correspond to observed values.

**FIG 3:**
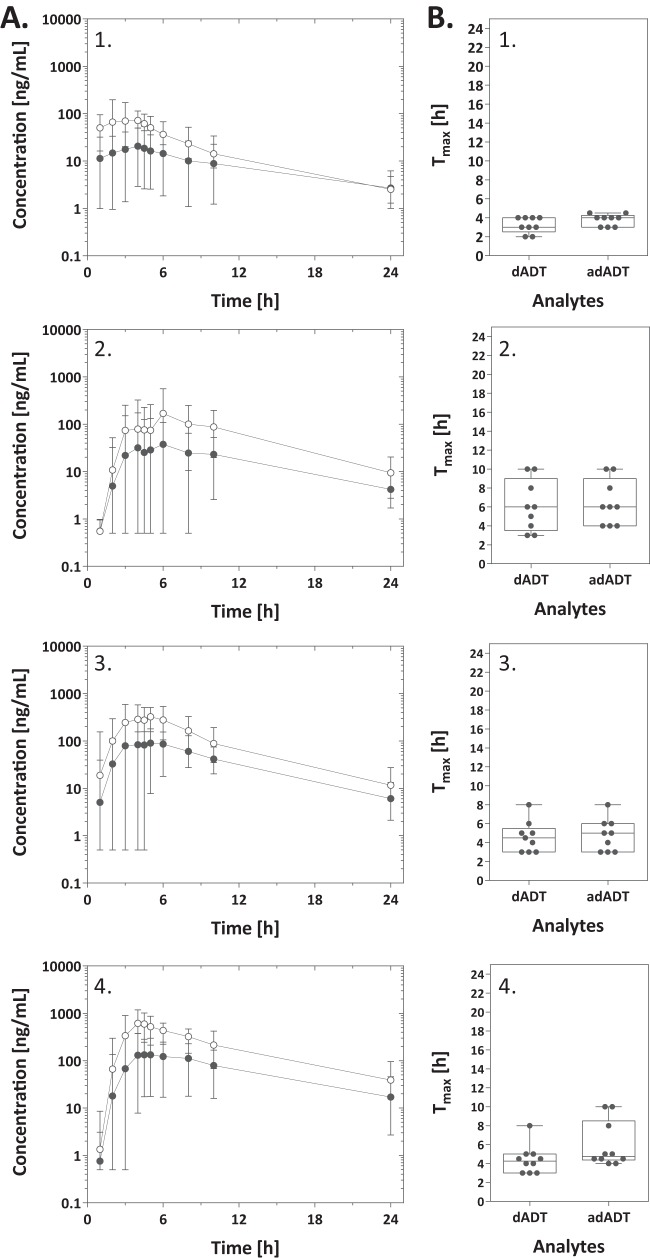
(A) Mean plasma concentration-time profiles for dADT (○) and adADT (●) following oral doses of one-half of a 50-mg tablet (*n* = 9) (A1), one 50-mg tablet (*n* = 9) (A2), two 50-mg tablets (*n* = 9) (A3), and four 50-mg tablets (*n* = 10) (A4) of tribendimidine. The mean concentrations and the ranges are illustrated. (B) Box plots of the *T*_max_ values for dADT and adADT (25 mg tribendimidine [B1], 50 mg tribendimidine [B2], 100 mg tribendimidine [B3], and four 50-mg tablets of tribendimidine [B4]). The upper and lower limits of the boxes correspond to the interquartile range, the value in the middle corresponds to the median, and the limits of the whiskers correspond to the range. Gray dots correspond to observed values.

Mean plasma concentration-time profiles for dADT and adADT in studies 1 and 2 are illustrated in Fig. 2 and 3, respectively. As expected, tribendimidine itself was not detected in the circulatory system. Twelve percent of dADT and 17% of adADT plasma samples could not be quantified due to concentrations from mainly early sampling time points being below the LLOQ. dADT maximal plasma concentrations of treatments with 200-mg tablets (dosages of 200 to 600 mg in study 1) ranged between 237 and 2,517 ng/ml, with a median of 744 ng/ml (IQR, 562 to 1,098 ng/ml) for the 200-mg dose, 1,398 ng/ml (IQR, 1,254 to 1,558 ng/ml) for the 400-mg dose, and 1,351 ng/ml (IQR, 1,294 to 1,561 ng/ml) for the 600-mg dose (Table 2). PK parameters calculated for adADT and from blood concentrations, which were very similar to plasma parameters, are presented in Table 2. In study 2, maximal plasma concentrations of dADT and adADT of 60 to 1,185 ng/ml and 3 to 375 ng/ml, respectively, were achieved with 25- to 200-mg treatments (median values and IQRs are presented in Table 3). The *T*_max_ of dADT was observed at between 2 and 24 h for treatments of 200 to 600 mg using 200-mg tablets (Table 2 and Fig. 2). adADT concentrations reached peak levels later than did dADT concentrations, as expected for a metabolite. The rate of absorption of 50-mg tablets was higher than that of 200-mg tablets, with less variability in *T*_max_ being observed between individuals (Fig. 3). The fastest absorption was observed for patients treated with 25 mg, most likely due to breaking and thus destruction of the enteral coating of the 50-mg tablets. Overall, the median *t*_1__/2_ values calculated over all subjects were 4 h (IQR, 3 to 5 h) and 5 h (IQR, 4 to 6 h) for dADT and adADT, respectively. No difference in *t*_1__/2_ values of dADT was observed between the two studies. However, the *t*_1/2_ of adADT was somewhat longer in study 1 than in study 2 (median of 6 h versus 4 h, respectively). Median dADT drug clearance (CL/*F*) values for 200-mg (study 1) and 50-mg (study 2) tribendimidine tablets were comparable, with 27 liters/h in study 1 and 35 liters/h in study 2. Mean residence times of 9.3 h and 13.0 h were observed for treatments using 200-mg tablets of dADT and adADT, respectively. Shorter MRTs of 7.6 h for dADT and 9.2 h for adADT were calculated in the case of treatments using 50-mg tablets.

**TABLE 2:** PK parameters of dADT and adADT following treatment with 200 mg, two 200-mg tablets, and three 200-mg tablets of tribendimidine determined in plasma and blood^*a*^

Analyte and PK parameter	Median value for treatment (IQR)								
	200 mg			400 mg			600 mg		
	Plasma	Blood	*N*^*b*^	Plasma	Blood	*N*^*b*^	Plasma	Blood	*N*^*b*^
dADT									
*C*_max_ (ng/ml)	744 (562–1,098)	694 (479–1,034)	13	1,398 (1,254–1,558)	1,205 (1,152–1,392)	9	1,351 (1,294–1,561)	1,142 (1,086–1,415)	9
*T*_max_ (h)	5 (4–8)	5 (4–8)	13	8 (6–10)	8 (5–10)	9	6 (4–8)	6 (4–8)	9
AUC_0–24_ (h · ng/ml)	5,888 (4,793–7,211)	5,387 (3,879–6,280)	13	11,668 (9,792–13,077)	9,857 (8,238–11,060)	9	12,402 (11,170–14,860)	10,312 (9,173–13,322)	9
AUC_∞_ (h · ng/ml)	6,459 (5,658–7,802)	6,181 (5,220–7,543)	10	12,044 (11,055–13,910)	10,179 (9,861–11,317)	5	14,003 (12,309–15,555)	11,830 (10,567–13,491)	6
AUC_0–24_ (h · ng · ml^−1^ · kg^−1^ body wt)	103 (89–129)	100 (82–117)	13	233 (204–267)	197 (172–229)	9	267 (200–316)	223 (173–321)	9
AUMC_∞_ (h^2^ · ng/ml)	62,002 (48,859–79,288)	57,812 (44,973–81,027)	10	116,831 (94,233–136,019)	105,024 (78,643–111,425)	5	120,004 (107,458–170,864)	104,133 (94,355–140,272)	6
MRT (h)	10 (8–10)	10 (8–11)	10	10 (8–10)	10 (8–10)	5	9 (8–11)	9 (9–11)	6
*t*_1/2_ (h)	4 (3–5)	4 (3–5)	10	4 (4–5)	4 (4–5)	5	4 (4–5)	4 (4–5)	6
CL/*F* (liters/h)	24 (20–28)	25 (21–30)	10	26 (23–28)	31 (28–32)	5	34 (30–38)	40 (35–44)	6
adADT									
*C*_max_ (ng/ml)	45 (34–142)	53 (38–152)	13	216 (56–398)	223 (56–423)	9	345 (200–427)	347 (191–422)	9
*T*_max_ (h)	8 (6–10)	8 (6–9)	13	10 (8–10)	9 (8–10)	9	8 (6–10)	8 (6–8)	9
AUC_0–24_ (h · ng/ml)	471 (323–1,270)	482 (334–1,357)	13	2,194 (738–3,972)	2,295 (753–4,198)	9	3,718 (2,474–4,514)	3,691 (2,456–5,024)	9
AUC_∞_ (h · ng/ml)	574 (489–1,387)	637 (542–1,480)	9	1,654 (917–2,853)	1,760 (1,013–2,994)	4	3,600 (2,456–4,571)	3,560 (2,326–4,436)	5
AUC_0–24_ (h · ng · ml^−1^ · kg^−1^ body wt)	11 (7–24)	12 (8–25)	13	39 (14–76)	42 (15–81)	9	70 (47–81)	69 (46–87)	9
AUMC_∞_ (h^2^ · ng/ml)	10,177 (6,632–14,693)	11,289 (7,001–15,664)	9	18,667 (14,880–29,820)	21,393 (17,953–31,842)	4	41,272 (31,947–52,127)	40,475 (29,585–48,942)	5
MRT (h)	14 (11–16)	13 (11–16)	9	12 (11–14)	12 (11–15)	4	11 (11–13)	11 (11–13)	5
*t*_1/2_ (h)	6 (5–8)	6 (5–9)	9	6 (5–7)	6 (5–8)	4	6 (6–6)	6 (6–6)	5

a AUC_∞_, AUMC_∞_, MRT, CL/*F*, and *t*_1/2_ were calculated only when three or more observations were available in the drug elimination phase.

b *N*, no. of participants included in the analysis.

**TABLE 3:** PK parameters of dADT and adADT following treatment with one-half of a 50-mg tablet, one 50-mg tablet, two 50-mg tablets, and four 50-mg tablets of tribendimidine determined in plasma^*a*^

Analyte and PK parameter	Median value for treatment (IQR)							
	25 mg		50 mg		100 mg		200 mg	
	Plasma	*N*^*b*^	Plasma	*N*^*b*^	Plasma	*N*^*b*^	Plasma	*N*^*b*^
dADT								
*C*_max_ (ng/ml)	68 (62–88)	9	252 (230–327)	9	508 (372–566)	9	701 (543–789)	10
*T*_max_ (h)	3 (3–4)	9	6 (4–8)	9	4 (3–5)	9	4 (3–5)	10
AUC_0–24_ (h · ng/ml)	463 (397–522)	9	1,238 (1,179–1,439)	9	2,448 (2,065–2,661)	9	4,406 (3,990–5,380)	10
AUC_∞_ (h · ng/ml)	485 (410–535)	9	1,249 (1,165–1,435)	7	2,475 (2,111–2,749)	9	4,914 (4,184–5,640)	10
AUC_0–24_ (h · ng · ml^−1^ · kg^−1^ body wt)	8 (7–9)	9	25 (22–35)	9	49 (41–60)	9	81 (72–98)	10
AUMC_∞_ (h^2^ · ng/ml)	2,882 (2,624–3,557)	9	10,075 (8,619–13,058)	7	16,803 (12,000–20,750)	9	36,290 (34,929–58,238)	10
MRT (h)	6 (6–7)	9	8 (7–9)	7	7 (6–8)	9	8 (8–9)	10
*t*_1/2_ (h)	4 (4–5)	9	3 (3–4)	7	4 (3–5)	9	4 (4–5)	10
CL/*F* (liters/h)	40 (37–48)	9	31 (28–34)	7	32 (29–37)	9	32 (28–37)	10
adADT								
*C*_max_ (ng/ml)	26 (6–29)	9	53 (13–82)	9	117 (88–180)	9	144 (46–243)	10
*T*_max_ (h)	4 (3–4)	9	6 (4–8)	9	5 (3–6)	9	5 (4–7)	10
AUC_0–24_ (h · ng/ml)	140 (29–222)	9	395 (112–398)	9	796 (706–993)	9	1,611 (440–2,228)	10
AUC_∞_ (h · ng/ml)	165 (48–239)	9	406 (196–655)	6	816 (715–1,043)	9	2,057 (1,571–2,467)	8
AUC_0–24_ (h · ng · ml^−1^ · kg^−1^ body wt)	3 (1–4)	9	8 (2–10)	9	17 (13–21)	9	28 (8–43)	10
AUMC_∞_ (h^2^ · ng/ml)	1,354 (491–2,167)	9	3,577 (1,979–6,969)	6	6,589 (5,186–9,883)	9	20,245 (13,741–24,635)	8
MRT (h)	9 (7–9)	9	9 (8–10)	6	9 (7–9)	9	9 (9–10)	8
*t*_1/2_ (h)	5 (4–6)	9	4 (4–5)	6	4 (4–5)	9	4 (4–5)	8

a AUC_∞_, AUMC_∞_, MRT, CL/*F*, and *t*_1/2_ values were calculated only when three or more observations were available in the drug elimination phase.

b *N*, no. of participants included in the analysis.

**FIG 4:**
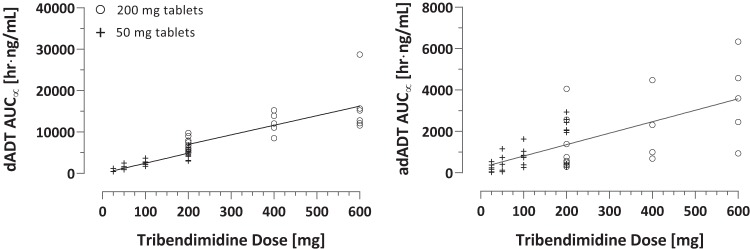
Correlation of single ascending doses of tribendimidine and AUC_∞_ of dADT and adADT. A difference in the relationship between AUC∞ and dose by tablet formulation was evident for dADT but not for adADT. In the case of dADT, the correlation coefficients calculated for 50-mg and 200-mg tablets were 0.83 (25 to 200 mg) and 0.56 (200 to 600 mg), respectively. The correlation coefficient calculated for adADT was 0.46 (25 to 600 mg).

A correlation of ascending doses of tribendimidine and the AUC_∞_ was observed for both dADT (*R*^2^ = 0.83 for 25 to 200 mg; *R*^2^ = 0.56 for 200 to 600 mg) and adADT (*R*^2^ = 0.46 for 25 to 600 mg) (Fig. 4). For dADT, there was evidence of a difference in the relationship between AUC_∞_ and dose by tablet formulation (interaction *P* value of 0.05), with results being consistent with dose proportionality for the 50-mg (90% CI, 0.95 to 1.15) but not the 200-mg (90% CI, 0.54 to 0.98) tablets. For adADT, overall dose proportionality could not be concluded (90% CI, 0.88 to 1.48, adjusting for tablet), and there was no evidence of a difference by tablet (interaction *P* = 0.91). Adjustment for weight led to similar results. The level of dADT exposure (AUC_∞_) was significantly higher in subjects treated with one 200-mg tablet than in those treated with four 50-mg tablets (*P* = 0.015 by a two-tailed Mann-Whitney U test). No significant difference could be detected for adADT exposure (AUC_∞_) between these two treatment groups (*P* = 0.37).

### Drug dissolution.

**FIG 5:**
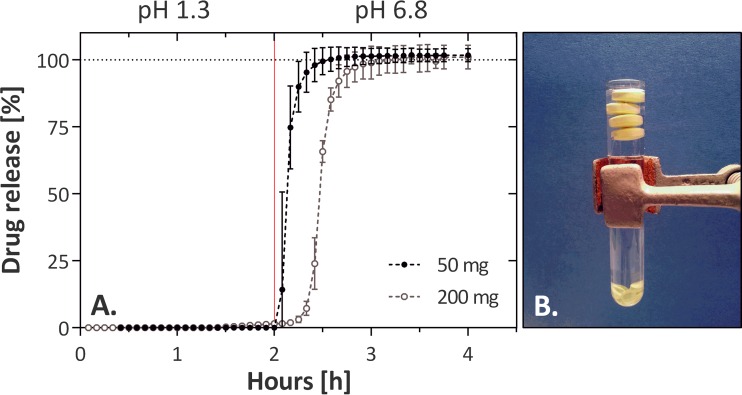
(A) Drug dissolution experiment with tribendimidine 200- and 50-mg tablets (*n* = 3). The mean drug release and 1.96× standard deviations are illustrated. Enteric coating prevents dissolution (<1.6%) of the tablets in acid medium (pH 1.3 at 0 to 2 h). Fast dissolution occurs at pH 6.8 (after 2 h), and the dissolution of the 50-mg tablets is marginally faster than that of the 200-mg tablets. (B) Image of 200-mg and 50-mg tablets in a test tube filled with acidified water. The 200-mg tablets float on top of the surface, whereas the 50-mg tablets sink to the bottom of the tube.

Drug dissolution experiments were carried out to better understand the differences in the interindividual absorption rates observed following treatment with 200-mg tablets (Fig. 5). Less than 1.6% of drug was released for both drug formulations (50- and 200-mg tablets) when incubated for 2 h at pH 1.3, indicating stability of the enteric coating. At pH 6.8, both tablet formulations released tribendimidine immediately. Fifty-milligram tablets achieved 100% drug release ∼1 h earlier than did 200-mg tablets. Overall, the variability of drug dissolution within the tablet formulations was small. The 200-mg tablets floated immediately to the top of the water surface area, while the 50-mg tablets sank to the bottom of the test tube (Fig. 5).

### Pharmacokinetic and pharmacodynamic relationship.

**FIG 6:**
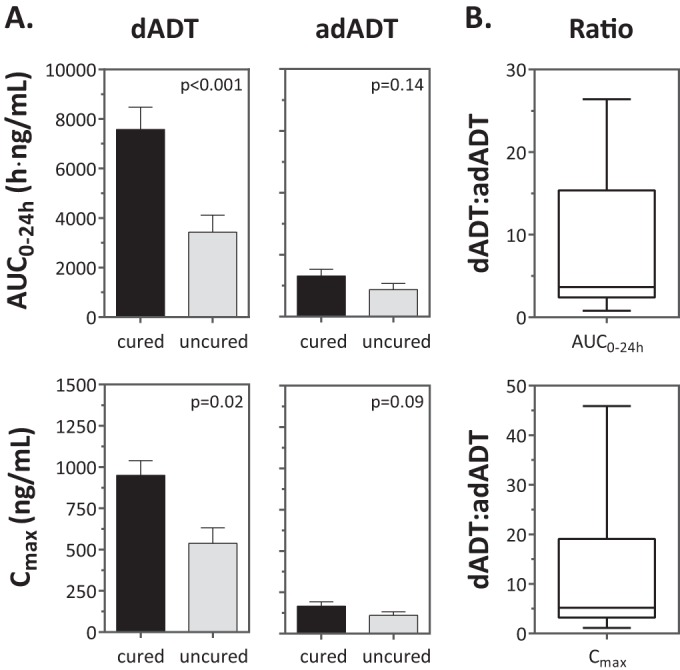
(A) Relationship between treatment outcome (cured/uncured) and the *C*_max_ and AUC_0–24_ values for dADT and adADT. The bars correspond to the means, and the error bars correspond to the standard errors. (B) Box plots of the AUC_0–24_ and *C*_max_ ratios of dADT to adADT show the variability of dADT metabolism observed in the study population. The upper and lower limits of the boxes correspond to the interquartile range, the value in the middle corresponds to the median, and the limits of the whiskers correspond to the range.

*In vitro* results demonstrated that dADT is very fast acting against O. viverrini, with a 90% effective concentration (EC_90_) of 75 ng/ml after 3 h of incubation (data not shown). adADT is not active *in vitro* at the concentrations reached following treatments with high tribendimidine dosages (600 mg) and therefore most likely does not contribute to the activity. The observed dADT *C*_max_ values were above the EC_90_ value even at low tribendimidine dosages (50 mg). Cured patients showed significantly higher *C*_max_ (*P* = 0.02) and AUC_0–24_ (*P* < 0.001) values for dADT than did uncured patients, while no significant relationship for *C*_max_ (*P* = 0.09) and AUC_0–24_ (*P* = 0.14) was observed for adADT (Fig. 6A). The median ratio of the AUC_0–24_ of dADT to the AUC_0–24_ of adADT was 3.7, with a range of 0.8 to 26.4, indicating substantial variations in the acetylation rates of the study population (Fig. 6B). A similar analysis of the ratios of dADT to adADT using AUC_∞_ (median of 3.1 and range of 0.8 to 21.1) or *C*_max_ (median of 5.2 and range of 1.1 to 45.9) revealed a comparable variability. The frequency distribution of the dADT/adADT ratios is skewed to the right in the case of all three parameters, which implies that most of the participants appear to have a fast-acetylator phenotype. We found weak evidence to suggest that patients with a higher acetylation rate, which corresponds to a lower dADT AUC_0–24h_/adADT AUC_0–24h_ ratio, were less likely to be cured than patients showing a lower acetylation rate (*P* = 0.13; odds ratio for not being cured, 0.92 [95% CI, 0.84 to 1.02]). Similar results were obtained by using AUC_∞_ or *C*_max_ values.

## DISCUSSION

Infections with helminths remain a pressing global health problem affecting billions of people worldwide, mainly in tropical and subtropical areas. To date, limited attention has been paid to developing and optimizing alternative drugs for their treatment. Tribendimidine, characterized by a broad spectrum of activity against nematode and trematode infections, might prove to be a useful alternative to the few existing drugs that are threatened by the rise of drug resistance (25, 26). Despite tribendimidine having been marketed in China for several years, little is known about its PK or its PD behavior against many helminths, including O. viverrini (9). Given current interest in obtaining FDA registration for tribendimidine and thus the ultimate use of the drug on a large scale, it is crucial to acquire further knowledge. In the present study, we assessed for the first time the drug disposition of tribendimidine using ascending dosages with corresponding treatment efficacy.

To adequately assess the PK of drugs for helminth and other tropical diseases, studies need to be carried out with infected populations, which brings many challenges. Helminth diseases occur mostly in rural regions often far away from hospitals offering appropriate infrastructure (27). This warrants the use of reliable and appropriate technologies, such as DBS, which are superior to traditional venous blood collection in these settings, since, among other advantages, hospitalization, sample centrifugation, and freezing are not required (28–30). We demonstrate that the observed mean bias between plasma and blood or DBS samples was <12% for the tribendimidine metabolites dADT and adADT, which implies that both analytes partition equally into erythrocytes and the plasma fraction. The clinical relevance of this bias is not known, yet tribendimidine has a rather wide therapeutic index considering efficacy and safety (16). However, 95% limits of agreement of plasma and DBS concentrations are rather wide, so a comparison of a single value might be imprecise. In addition, it was not taken into account that multiple determinations per individual were included in the analysis. Nevertheless, the agreement of DBS and plasma data was better for secondary PK parameters (AUC_0–24_), which made use of multiple measurements per person (Fig. 1; see also Fig. S1 in the supplemental material). Data from the literature suggest that methodological improvements of the analytical method, such as, e.g., spraying the internal standard onto the blood spots or analyzing more than one DBS per time point, might further increase the accuracy and precision of the DBS technique (31, 32).

Our PK study in opisthorchiasis patients in Lao People's Democratic Republic was conducted in two parts. The first study assessed doses of 200 to 600 mg using 200-mg tablets, which are licensed in China. Given the high efficacy observed with these dosages, a second study was conducted to evaluate 25 to 200 mg tribendimidine using 50-mg tablets (16).

Surprisingly, to date, only two studies have evaluated the disposition of tribendimidine in humans (12, 33). In these studies, 400 mg tribendimidine was given to healthy volunteers (12). PK studies with 50-mg tablets have not previously been conducted. The observed *C*_max_ and AUC_0–24_ of dADT in plasma were about 2 to 3 times lower in those studies than in our study. However, comparison of data from the three studies is difficult, as different sampling designs and PK models were used. In addition, our study participants were not healthy but infected with O. viverrini. Clinical parameters indicated that several participants in our study had mild renal insufficiency, and half of the patients enrolled had elevated levels of liver enzymes, which may explain the increased dADT disposition. Note that secondary PK parameters estimated by the noncompartmental analysis described here were compared with data from compartmental PK analyses and data in the literature by Vanobbergen and colleagues (34).

The time to maximal plasma concentrations in our study was very variable and considerably delayed compared to those reported in previous studies (12, 33). Drug dissolution experiments demonstrated that the currently available 200-mg tribendimidine tablets have a low specific weight. They immediately float in water and therefore can be expected to have a prolonged residence time within the stomach (35). In combination with an enteric coating, this might lead to delayed drug absorption, as almost no drug is released until it reaches the intestine. The nonfasted state of our study participants might further prolong gastric retention and extend drug absorption (36). Of note, volunteers in the study by Yuan and colleagues (12) fasted for 10 h before drug intake, which might explain the 2-fold-lower *T*_max_ in their study than in ours. The MRT of the 50-mg tablets was ∼2 h shorter than that of the 200-mg tablets, in line with the nonfloating characteristics and faster drug release of the 50-mg tablets. In addition, we observed that breaking of the enteric coating (as for the 25-mg treatments) resulted in uniform and fast absorption. dADT could be detected in the body even if tablets were split, which suggests that dADT is not destroyed in the stomach; hence, the enteric coating is not needed to achieve an effect against O. viverrini.

Dose proportionality for dADT was observed from 25 to 200 mg for treatments with 50-mg tablets but not for higher dosages (200 to 600 mg) with 200-mg tablets. More data would be needed to evaluate whether the tapering off arises from differences in drug formulations or if there is saturation at high doses.

The rates of acetylation of dADT to the inactive adADT varied considerably between individuals. In our study population, most of the participants appeared to have a relatively high metabolic turnover of dADT to adADT. This is expected, as 90% of the Asian population exhibit a fast-NAT-2 phenotype (37). However, the dADT AUC_0–24h_/adADT AUC_0–24h_ ratio might be influenced not only by metabolic turnover but also by delayed drug absorption (which would have required longer follow-up measurements). Still, calculations based on AUC_∞_ or *C*_max_ also revealed very variable dADT/adADT ratios within the study population. Overall, the rate of dADT metabolism seems to influence treatment efficacy, since dADT and not adADT is active against O. viverrini. Population PK studies are ongoing to determine the influence of other covariates given that the PK/PD relationship might be multifactorial. Further studies are necessary since ethnic differences in NAT-2 polymorphisms are considerable (38) and ultimately might influence the treatment safety of tribendimidine and outcome against O. viverrini infections. This issue is also pertinent given that tribendimidine may be widely used against O. felineus and soil-transmitted helminth infections outside Asia in the near future; this issue should be carefully studied.

In conclusion, we have described for the first time the disposition of different dosages of tribendimidine in opisthorchiasis patients using two different tablet formulations. Differences in the disposition of tribendimidine were observed in opisthorchiasis patients compared to healthy volunteers. We found that cured patients showed significantly higher *C*_max_ and AUC_0–24h_ values for dADT than uncured patients. Our study confirmed that adADT has marginal activity, since we observed no evidence of an association between cure and adADT exposure. Further PK studies in opisthorchiasis patients should be conducted once the correct dose and formulation have been established. In addition, the impact of the large differences between individuals in the rates of metabolic turnover of dADT to adADT should be studied in future clinical studies.

## Supplementary Material

Supplemental material
