# Supplementary material for: Single-Ascending-Dose Pharmacokinetic Study of Tribendimidine in Opisthorchis viverrini-Infected Patients
Source: Antimicrob Agents Chemother. 2016 Sep 23;60(10):5705–15. doi: 10.1128/AAC.00992-16 (PMC5038241; doi:10.1128/AAC.00992-16)
Supplement: Supplemental material [file supp_60_10_5705__index.html]

Single-Ascending-Dose Pharmacokinetic Study of Tribendimidine in Opisthorchis viverrini-Infected Patients — Supplemental material 

# Single-Ascending-Dose Pharmacokinetic Study of Tribendimidine in Opisthorchis viverrini-Infected Patients

## Supplemental material

- Supplemental file 1 -

  Table S1 and Figure S1

  PDF, 910K
