# Supplementary material for: Single-Ascending-Dose Pharmacokinetic Study of Tribendimidine in Opisthorchis viverrini-Infected Patients
Source: Antimicrob Agents Chemother. 2016 Sep 23;60(10):5705–15. doi: 10.1128/AAC.00992-16 (PMC5038241; doi:10.1128/AAC.00992-16)
Supplement: Supplemental material [file AAC.00992-16_zac010165542so1.pdf]

Supplementary files

Table S1. Inter-assay accuracy and precision of dADT and adADT QC samples determined in DBS, plasma, and blood.

Analyte	Nominal concentration	DBS			Plasma			Blood		
		Accuracy ± CV [%]	N	Study	Accuracy ± CV [%]	N	Study	Accuracy ± CV [%]	N	Study
**dADT**	1500	95.2 ± 10.2	12	1						
	750	99.6 ± 3.7	12	2	99.2 ± 3.4	72	1,2	100.2 ± 5.9	24	1
	300	98.1 ± 3.2	12	1						
	30	102.2 ± 7.5	24	1,2	101.7 ± 2.9	72	1,2	103.3 ± 7.4	24	1
	10	97.8 ± 7.5	12	1						
	3	103.1 ± 9.7	11	2	101.0 ± 4.7	72	1,2	104.0 ± 4.0	24	1
	1	101.8 ± 10.4	11	2	97.7 ± 9.6	72	1,2	106.3 ± 6.2	24	1
**adADT**	1500	94.6 ± 12.7	12	1						
	750	101.1 ± 5.8	12	2	96.3 ± 4.2	72	1,2	102.2 ± 2.4	24	1
	300	98.7 ± 5.1	12	1						
	30	105.1 ± 9.5	24	1,2	101.5 ± 3.7	72	1,2	103.4 ± 5.9	24	1
	10	98.1 ± 10.5	12	1						
	3	103.2 ± 5.9	11	2	100.5 ± 4.4	72	1,2	102.4 ± 4.6	24	1
	1	108.1 ± 15.7	11	2	98.2 ± 8.5	72	1,2	105.5 ± 7.9	24	1

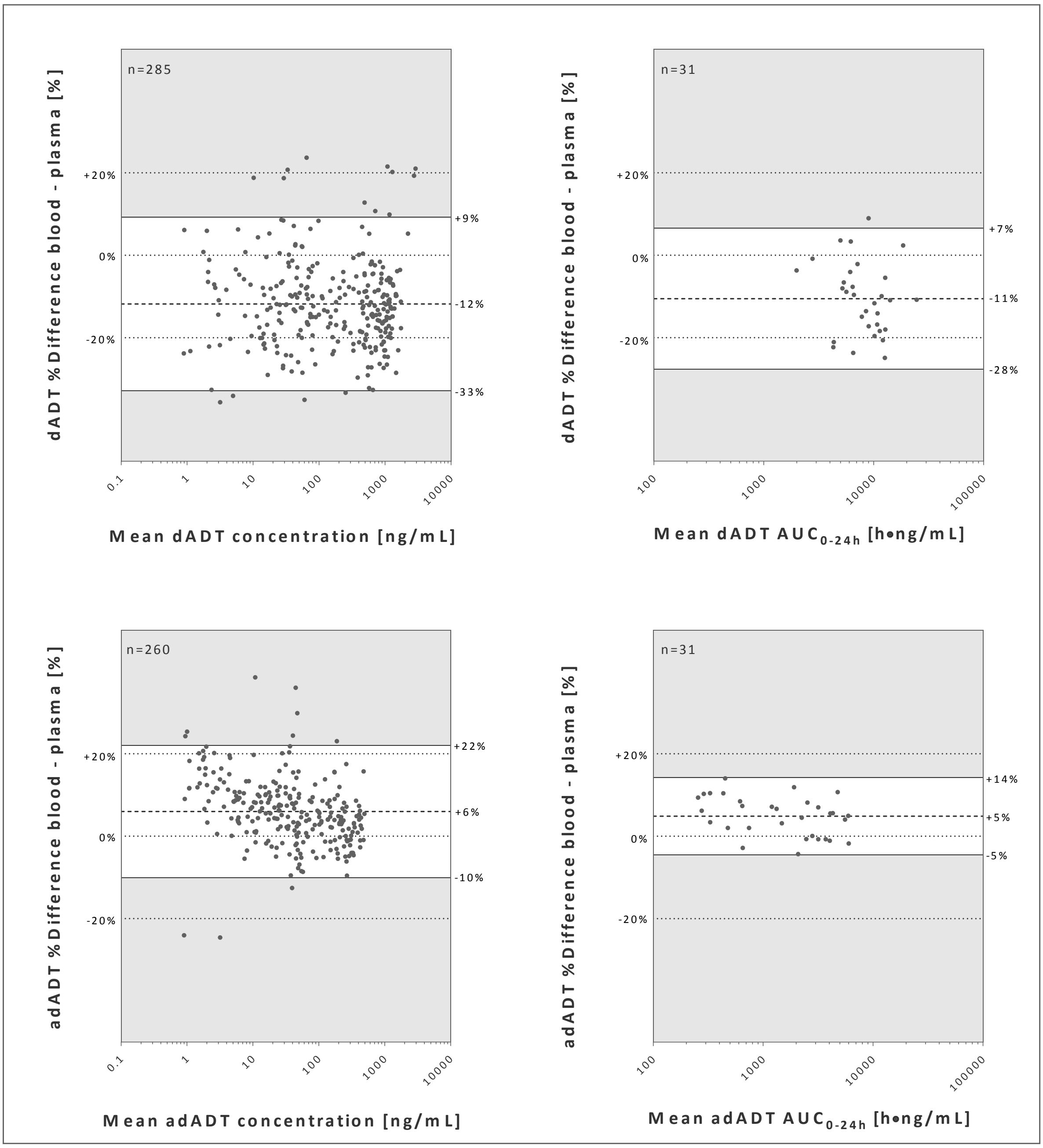

Figure S1. Bland-Altman plot of concentrations and $AUC_{0\text{-}24h}$ measured in blood and plasma (%Difference vs mean). The AUC was calculated from time point zero to the time point of last quantifiable concentration. Only concentration data which were available for both biofluids were included. The dashed line in dark grey illustrates the mean %Difference of blood to plasma. The white area defines the 95% limits of agreement. ±20% bias from zero are marked in the plots as they refer to limits applied for cross-validation of two bioanalytical methods. Grey dots correspond to observed values.
